# Linear Binary Classifier to Predict Bacterial Biofilm Formation on Polyacrylates

**DOI:** 10.1021/acsami.2c23182

**Published:** 2023-03-07

**Authors:** Leonardo Contreas, Andrew L. Hook, David A. Winkler, Grazziela Figueredo, Paul Williams, Charles A. Laughton, Morgan R. Alexander, Philip M. Williams

**Affiliations:** † School of Pharmacy, University of Nottingham, Nottingham NG7 2RD, United Kingdom; ‡ Monash Institute of Pharmaceutical Sciences, Monash University, Parkville, Victoria 3052, Australia; § Department of Biochemistry and Genetics, La Trobe Institute for Molecular Science, La Trobe University, Bundoora, Victoria 3086, Australia; ∥ School of Computer Science, University of Nottingham, Nottingham NG8 1BB, United Kingdom; ⊥ National Biofilms Innovation Centre and Biodiscovery Institute, School of Life Sciences, University of Nottingham, Nottingham NG7 2RD, United Kingdom

**Keywords:** bacterial attachment, healthcare-associated infections, machine learning, classification, polyacrylates

## Abstract

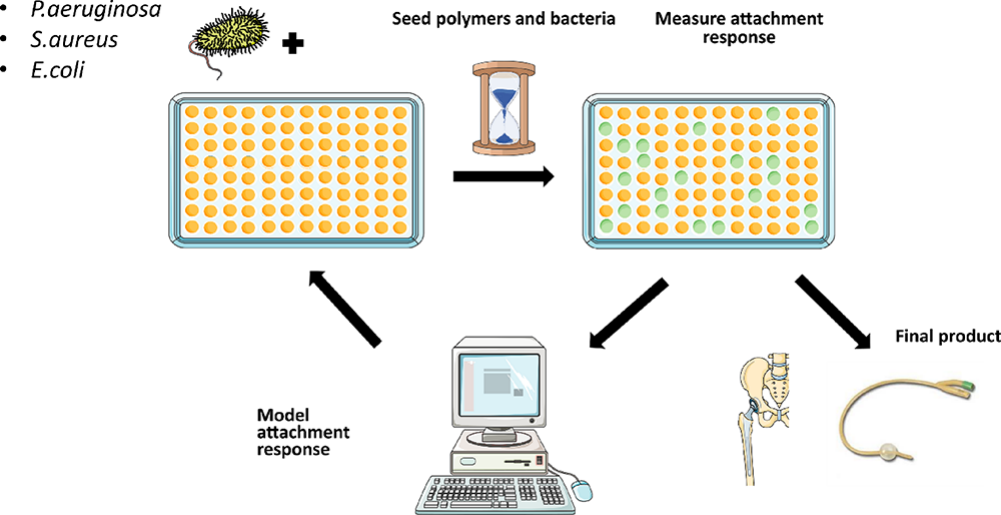

Bacterial infections are increasingly problematic due to the rise of antimicrobial resistance. Consequently, the rational design of materials naturally resistant to biofilm formation is an important strategy for preventing medical device-associated infections. Machine learning (ML) is a powerful method to find useful patterns in complex data from a wide range of fields. Recent reports showed how ML can reveal strong relationships between bacterial adhesion and the physicochemical properties of polyacrylate libraries. These studies used robust and predictive nonlinear regression methods that had better quantitative prediction power than linear models. However, as nonlinear models’ feature importance is a local rather than global property, these models were hard to interpret and provided limited insight into the molecular details of material–bacteria interactions. Here, we show that the use of interpretable mass spectral molecular ions and chemoinformatic descriptors and a linear binary classification model of attachment of three common nosocomial pathogens to a library of polyacrylates can provide improved guidance for the design of more effective pathogen-resistant coatings. Relevant features from each model were analyzed and correlated with easily interpretable chemoinformatic descriptors to derive a small set of rules that give model features tangible meaning that elucidate relationships between the structure and function. The results show that the attachment of *Pseudomonas aeruginosa* and *Staphylococcus aureus* can be robustly predicted by chemoinformatic descriptors, suggesting that the obtained models can predict the attachment response to polyacrylates to identify anti-attachment materials to synthesize and test in the future.

## 1 Introduction

Bacterial infections are a major problem in healthcare due largely to increasing antimicrobial resistance and larger numbers of patients with weakened or compromised immune systems. In 2002, it was estimated that, in the United States, almost 2 million patients suffered from healthcare-associated infections (HAIs) and 6% died.^1^ The estimated annual cost to hospitals was between US$28 billion and 45 billion.^2^ Two types of nosocomial infections were predominant: surgical site infections (SSI) with 22% of occurrence and urinary tract infections (UTIs) that account for a third of the cases.^1^ They represented more than half of all HAIs in the US outside of intensive care units (ICUs). A similar situation exists in the United Kingdom: In 2016/2017, an estimated 834,000 patients suffered from HAIs, and 28,500 of whom died (3.4%).^3^ Antimicrobial resistance (AMR) evolves when bacteria are subjected to the selection pressures by antibiotics and biocidal agents by drugs. Prolonged therapies, inappropriate prescriptions, self-medication, and overuse of antibiotics in agriculture have enabled the emergence of bacterial strains that are not susceptible to most or all antibiotic drugs.^4^ Therefore, new approaches are urgently needed to address the problem of HAIs. Preventing infection is clearly better than killing pathogens as the selective pressure to develop resistance is removed. Prevention is commonly achieved by modifying the surface of a medical implant^5,6^ by altering the surface chemistry^7,8^ or adsorbing/covalently coupling bactericidal molecules to the surface.^9,10^

After contact with a surface, biofilm formation occurs in three main stages—attachment, microcolony formation, and maturation. Initially, bacteria attach to a surface reversibly.^9^ Factors that promote attachment include surface hydrophobicity (the presence of a water layer can prevent bacteria from adhering to the surface)^10^ and a positively charged surface (most bacterial species are negatively charged).^11^ Rough and porous surfaces promote bacterial adhesion due to their greater surface area.^9,12^ Importantly, other surface biomaterial properties such as stiffness and topography may also play a role in bacterial attachment.^11^ Subsequently, bacteria attach irreversibly by excreting an extracellular matrix (ECM) that promotes surface adhesion and mature biofilm development.^13^ In vivo, adhesion can also be aided by interactions with the host blood and tissue proteins including fibronectin, fibrinogen, and thrombin, while albumin inhibits adhesion.^14^ The biofilm ECM is composed mostly of exopolysaccharides, proteins, and extracellular DNA^15^ that dramatically reduce immune responses and drug treatment efficacy by shielding bacteria from antimicrobials and the host immune system.^16^ When the bacterial biofilm has matured, cells disperse to the surrounding areas to seed sites for new biofilms to develop.^9^

The relationship between the multiple surface properties associated with initial bacterial attachment is complex and poorly understood, hampering de novo design of bacterium-resistant materials. It is known that the surface topography can modulate cell behavior; however, literature evidence suggests that the surface chemistry is the dominant factor.^17−20^

A useful way to analyze the complex relationships between material properties and biological responses is quantitative structure–activity relationship (QSAR) modeling.^21^ Several reports have elucidated factors that drive bacterial attachment to polymeric surfaces using partial least squares (PLS) regression^22^ or more complex machine learning (ML) methods. ML methods have been particularly successful in predicting the attachment response of *Staphylococcus aureus* (SA), *Pseudomonas aeruginosa* (PA), and uropathogenic *Escherichia coli* (UPEC) on mono- and polyacrylates.^23,24^ These studies focused on the quantitative prediction of bacterial attachment using a Bayesian regularized neural network (BRANN).^25^ Results showed that both computed molecular descriptors (from the commercial package Dragon^26^) and experimental mass spectral molecular ions contained information that was useful for predicting bacterial attachment of new polymers. However, these nonlinear regression models were difficult to interpret and do not provide simple design rules that a polymer chemist could use to synthesize improved biomaterials. Nonlinearity means that the importance of chemical features is local, not global, depending on where they are assessed.

Here, we adopted a simpler approach using logistic regression, a binary linear classifier^27^ that is more interpretable than the nonlinear regression models. The aim was to explain the role of key molecular features on the attachment of PA, SA, and UPEC to polyacrylates while still retaining most of the predictive power of the more complex nonlinear models. We separated polyacrylates into pro- and anti-attachment classes rather than use quantitative models of bacterial adhesion to the polymers. To further simplify the analysis, we studied the attachment response of these three different bacterial species separately. The polyacrylates used for this study were synthesized and incubated with three different suspensions of planktonic bacteria, and their chemical compositions were analyzed and characterized by time-of-flight secondary ion mass spectrometry (ToF-SIMS) as described in two experimental publications.^7,22^ The molecular ions obtained via ToF-SIMS contain information on the surface chemistry that bacteria would sense and respond to. They were used to train models together with 200 molecular descriptors obtained from the RDKit chemoinformatics Python library. Bacterial attachment response models for PA, SA, and UPEC were generated using ToF-SIMS data alone, chemoinformatic descriptors alone, or all features combined after applying several feature selection methods to reduce the model complexity and risk of overfitting. Models with good predictive capabilities were found for all three pathogens. Importantly, the most relevant features from each model were interpreted as a small number of simple design rules.

## 2 Experimental Section

### 2.1 Datasets

The datasets described below and Python code to process are provided in the Supporting Information. The pathogen attachment data consisted of two different datasets. One (denoted c496) consists of 496 homo- and co-polymeric acrylates, while the other (denoted h106) consists of 106 homo-polyacrylates. These two datasets were generated by Hook et al. and are described in experimental publications.^7,22^ Polymers were incubated with green fluorescent protein (GFP)-transformed PA (strain PAO1), SA (strain 8325-4), or UPEC (strain O6:K15:H31). The fluorescence intensity was strongly correlated with the number of bacteria remaining on the surface after incubation. The polymers were analyzed by ToF-SIMS for their surface chemical compositions. Since each polymer in the c496 dataset had several replicates, outliers (data point replicates that conflicted with the others) were detected and removed using the modified Thompson’s tau, as reported by Mikulskis et al.^24^ After outlier removal, the c496 dataset consisted of 492 polymers, while the h106 dataset contained 98 polymers. In addition, 200 descriptors were computed using the Python chemoinformatics library RDKit^28^ to increase the diversity of molecular information. We chose this package as binary classification is a simpler task than nonlinear regression and the RDKit is an accessible open-source package. When computing descriptors of co-polymers, cheminformatic descriptors were first computed for both monomeric components, and the resultant descriptor vector was the weighted mean of vectors of single components according to their ratio in the co-polymer as has been successful in prior studies. The Supporting Information contains the full list of descriptors used by the models (S-4).

### 2.2 Class Assignment and Training/Test Set Splitting

Unlike regression, which tries to predict quantitative attachment values, classification models find the best categorization of a dataset into defined classes (pro- or anti-attachment in the current case). Class labels are generated by setting a threshold value. Responses above the cut-off are categorized as “positives”; otherwise, they are labeled as “negatives”. In our case, all polymers with a fluorescence signal below the detection limit were given the label “0” (anti-attachment); otherwise, the label was set to “1” (pro-attachment). Since fluorescence data were collected from three different bacterial species, each polymer had three class labels.

We generated different datasets by combining c496 and h106 libraries or using the larger library (c496) alone. This was necessary because of the following.

(a) A poor class balance in some cases forced us to merge c496 and h106 samples to increase the representativity of minority class.
(b) c496 differed from h106 in its molecular character: the former was mainly made of co-polymers; the latter was exclusively made of homopolymers. This hampered the use of one dataset to predict the attachment response of polymers in the other and forced us to use either the larger set (c496) as the main dataset or the merged c496 and h106 datasets.
(c) ToF-SIMS ions in c496 did not follow the same distributions as those in h106. The ion peak values had dramatically different ranges. This prevented us from merging the two datasets using ToF-SIMS data. However, the use of RDKit descriptors for merged samples was still possible because computed descriptors consistently represent chemical structures with no bias.

Regardless of the dataset used, a fraction of the dataset (20% for PA and UPEC and 10% for SA because of poor class balance) was selected as a test set using a fixed random seed (preserving the same positive/negative class ratio). Model training and cross-validation were performed on the remaining 80 or 90% of data samples. The data sets were not balanced, so the training sets were resampled by randomly removing polymers belonging to the majority class until a 60/40 ratio of classes was obtained. However, the original class balance was not altered in the test set. All descriptors were standardized using the Z-score formula:
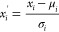
1where *x*_i_^′^ is the standardized descriptor vector, *x_i_* is the non-standardized descriptor vector in the *i*th column of the dataset, μ_*i*_ is its average value, and σ_*i*_ is its standard deviation. Data standardization is widely used, and it is common practice to avoid a dataset with large differences between the magnitudes of descriptors and to allow the learning algorithm to converge.^29^

Table 1 summarizes the dataset type, training and test set sample size, and class balance after majority class undersampling.

**Table 1:** Dimensions and Class Balance of the Datasets Used^a^

dataset	training set (positive, negative)	test set (positive, negative)
*P. aeruginosa* (c496)	144 (86,58)	99 (84,15)
*P. aeruginosa* (c496 + h106)	192 (115,77)	118 (99,19)
*S. aureus* (c496 + h106)	169 (101,68)	59 (52,7)
*E. coli* (c496)	389(156,233)	99 (39,60)
*E. coli* (c496 + h106)	472 (191,281)	118 (48,70)

a The table shows dataset sizes and class balance in terms of positive (first number in brackets) and negative (second number in brackets) samples for training and test sets.

### 2.3 Quantitative Modeling

Before training, the number of descriptors was reduced to discard highly correlated and low-diversity features. A correlation matrix using squared Pearson’s *r^2^* was computed to identify any highly correlated descriptors, and descriptors with low information contents (low variance) across the dataset were removed (S-3). Entropy can assume any value between 0 and 1, and high values reflect descriptors with a high amount of information. These two feature suppression criteria (multicollinearity and diversity) were applied at different thresholds (0.7, 0.8, and 0.9) each time to narrow the feature space down to a more manageable number of descriptors to save on computational cost and avoid overfitting. Both multicollinearity and entropy filters act as feature selection methods that are independent of the ML algorithm used.

After carrying out this first feature selection process, a wrapper method was also used to further reduce the size of the feature set in the final model. Wrapper methods are feature selection methods that use a learning algorithm for the feature selection process.^30,31^ We used sequential forward selection (SFS) rather than backward elimination because the modest number of samples in the dataset necessitated the models incorporating a relatively small number of features. A conservative rule of thumb suggests the use of only 1 feature for every 10 samples.^32^

Logistic regression (LR)^27^ was the machine-learning algorithm used inside the wrapper. It is a fast, simple, yet powerful linear classifier whose regularization type and strength can be tuned to avoid overfitting. A high regularization strength (the lower the value, the stronger) penalizes large coefficients during fitting, while by choosing an L1 penalty type over L2 causes the setting of many less relevant feature coefficients to zero, thus performing a sparse selection.^33^ An LR model was trained on each feature subset that the wrapper provided, and the performance was assessed through 10-fold cross-validation. The feature whose inclusion provided the highest cross-validation score was then incorporated into the updated set of descriptors. At the same time, the LR model was used to predict pathogen adhesion to the test set polymers. The optimal feature list was chosen on the basis of the highest and most consistent score across training, validation, and test phases. Performance was evaluated through sensitivity, specificity,^34^ and the Matthew’s correlation coefficient (MCC).^35^ Sensitivity and specificity can assume any values between 0 and 1. These three metrics indicate how accurately models classify positive and negative samples and are equal to zero for random prediction (null model). These three metrics are defined as follows
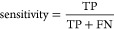
2
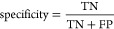
3
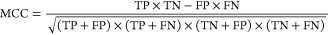
4where TP, TN, FP, and FN are the number of true positives, true negatives, false positives, and false negatives predicted by the model, respectively. The MCC ranges from −1 to 1 and is equal to zero if the model makes random predictions. It corrects performance overestimation in unbalanced datasets by lowering good scores if the class ratio is far from balanced, so it is a very useful metric to adopt with real-life classification datasets, which is similar to the F1 score or G mean.^36−38^

## 3 Results and Discussion

The results of LR modeling of data for the three pathogens are summarized in Table 2.

**Table 2:** Summary of the Classification Model Performance^a^

					training set	cross-validation	test set			
*N*	pathogen (model name)	R^2^ (diversity threshold)	regularizer	descriptor number (type)	MCC	MCC, mean, and SD (p value)	MCC (scrambled)	sens	spec	G mean
1	PA (c496 + h106)	0.9	L1, 10	22 (RDKit)	0.53	0.47 ± 0.12 (<10^–6^)	0.36 (0.12)	0.80	0.63	0.71
**2**	**PA (c496)**	**0.8**	**L1, 100**	**13 (RDKit)**	**0.54**	**0.51 *±* 0.24 (<10^–4^)**	**0.45 (.21)**	**0.82**	**0.73**	**0.77**
**3**	**PA (c496)**	**0.7**	**L1, 0.1**	**3(RDKit + ToF)**	**0.41**	**0.46 *±* 0.23 (<10^–4^)**	**0.44 (0.19)**	**0.85**	**0.67**	**0.75**
**4**	**PA (c496)**	**0.9**	**L1, 100**	**9 (ToF)**	**0.56**	**0.60 *±* 0.20 (<10^–5^)**	**0.48 (0.20)**	**0.76**	**0.87**	**0.81**
**5**	**SA(c496 + h106)**	**0.8**	**L2, 10**	**19 (RDKit)**	**0.64**	**0.65 *±* 0.18 (<10^–5^)**	**0.68 (0.21)**	**0.96**	**0.71**	**0.83**
**6**	**SA(c496 + h106)**	**0.7**	**L2, 0.1**	**4 (RDKit)**	**0.58**	**0.67 *±* 0.13 (<10^–7^)**	**0.57 (0.14)**	**0.92**	**0.71**	**0.81**
**7**	**UPEC (c496)**	**0.8**	**L1, 10**	**53 (ToF)**	**0.46**	**0.33 *±* 0.14 (<10^–4^)**	**0.41 (0.23)**	**0.64**	**0.77**	**0.70**
8	UPEC (c496)	0.9	L2, 100	24 (RDKit + ToF)	0.40	0.38 ± 0.14 (<10^–5^)	0.33 (0.16)	0.62	0.72	0.67

a The best models, which are used for subsequent feature interpretation, are shown in bold. Columns indicate the name of the model, threshold used for multicollinearity and entropy filtering, regularizing model, number, and type of descriptors, the training, cross-validation (*p* value for t-test for the score > 0), and test set MCC, sensitivity, specificity, and geometric mean.

**Figure 1:**
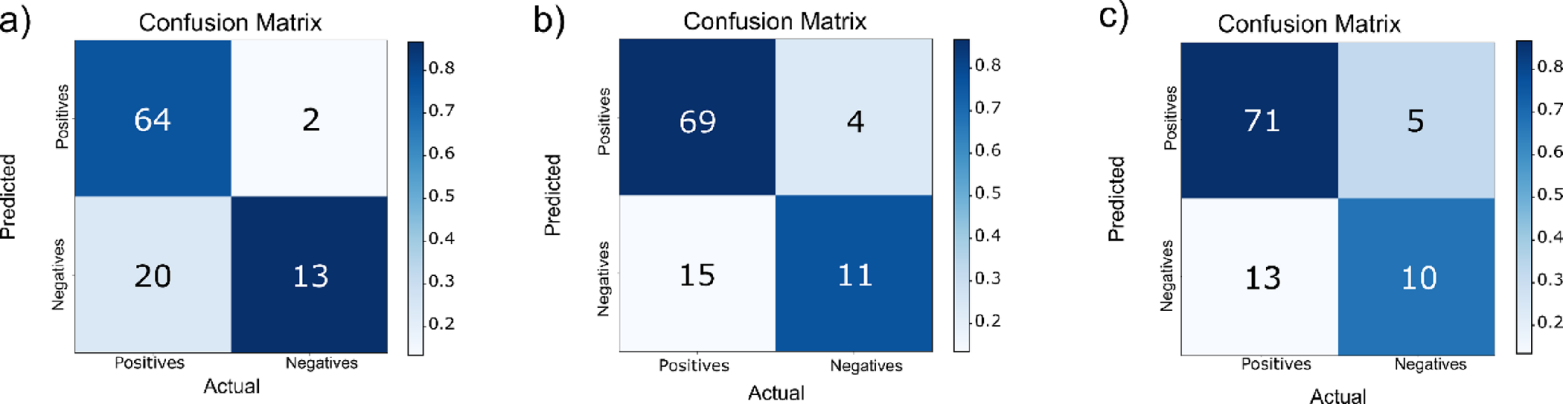
Confusion matrices for test set predictions for PA models. (a) Confusion matrix for PA-ToF (*p* < 0.00001). (b) Confusion matrix for the PA-RDKit (*p* < 0.0001). (c) Confusion matrix for PA-RDKit + ToF (*p* < 0.001).

All four PA models were statistically significant regardless of the type of descriptor and dataset used. The three models (Models 2–4) trained on the c496 dataset were similar and had higher prediction accuracies than Model 1 trained on both data sets. Model 4, using nine ToF-SIMS ion descriptors and trained on the c496 dataset, was selected as the best PA model based on a balance between the test set MCC and G mean and sparsity. However, Models 2, 3, and 4 were used for subsequent feature interpretation because of their similar predictive powers. Notably, Model 3 (using the c496 dataset with ToF-SIMS and RDKit descriptors) required only three features (two ToF-SIMS ion peaks and one RDKit descriptor). To ensure that the model performances were not due to chance, we performed a Fisher exact test^39,40^ on the confusion matrices of the models (Figure 1).

Statistical tests on the three best-performing PA models (Models 2, 3, and 4 of Table 2) provided a *p* value of <0.0001 for Model 2 (13 RDKit descriptors), a *p* value of <0.001 for Model 3 (2 ToF ions + 1 RDKit descriptor), and the best results for Model 4, which used nine ToF ions (*p* value < 0.00001). Although all three models passed the Fisher exact test, Model 2, using 13 RDKit descriptors, was the best compromise between the predictive power and interpretability.

For SA, two models with similar performance were generated, and both used RDKit descriptors and combined c496 and h106 datasets. One model used 19 features and the other only 4 to predict the attachment. The need to merge c496 and h106 datasets defined a larger domain of applicability for the models. However, the original poor class balance in the SA dataset resulted in negative samples being under-represented in the test set (whose class ratio was left untouched after splitting the dataset into training and test sets). Therefore, the test set MCC and G mean were more appropriate measures of prediction performance than the other metrics.

**Figure 2:**
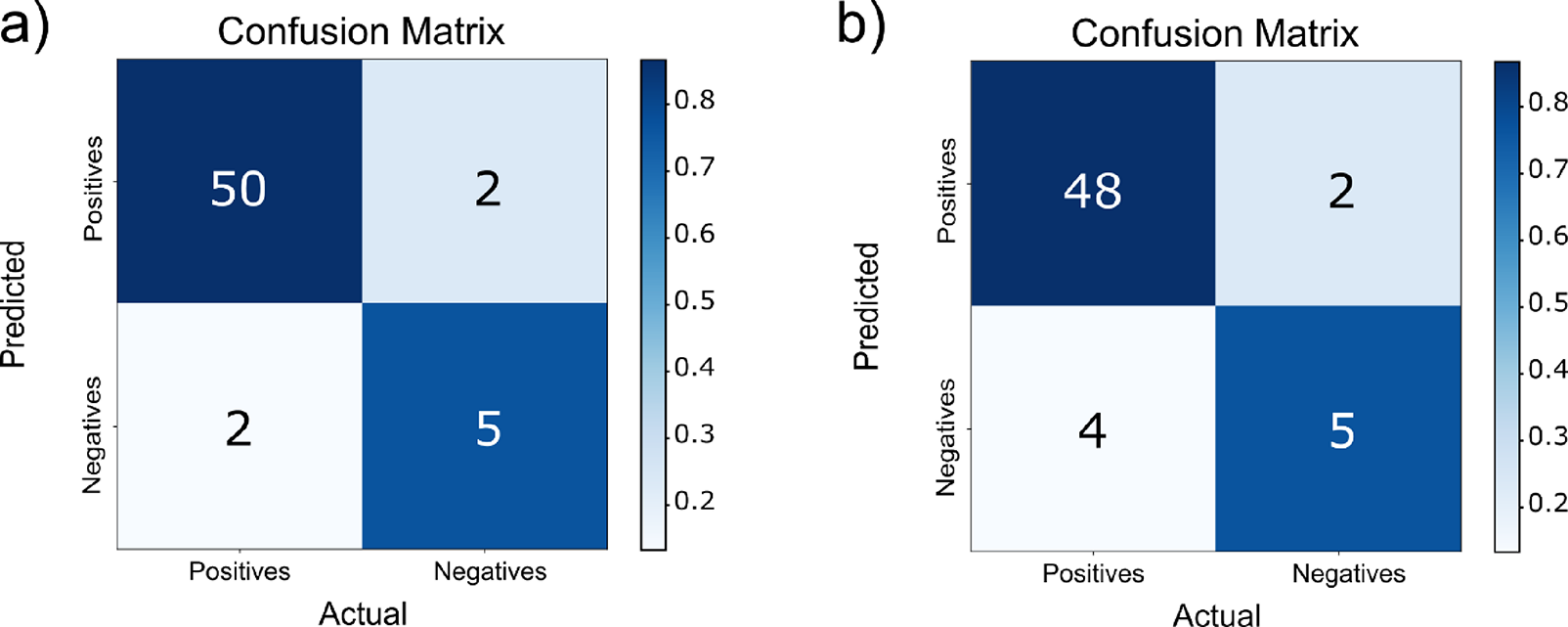
Confusion matrices for test set predictions of SA models. (a) SA-RDKit extended (*p* < 0.0001) and (b) SA-RDKit simple (*p* < 0.001).

SA models (Models 5 and 6 of Table 2) were significant with *p* values of <0.0001 and <0.001 (Fisher exact test), respectively (Figure 2a,b).

**Figure 3:**
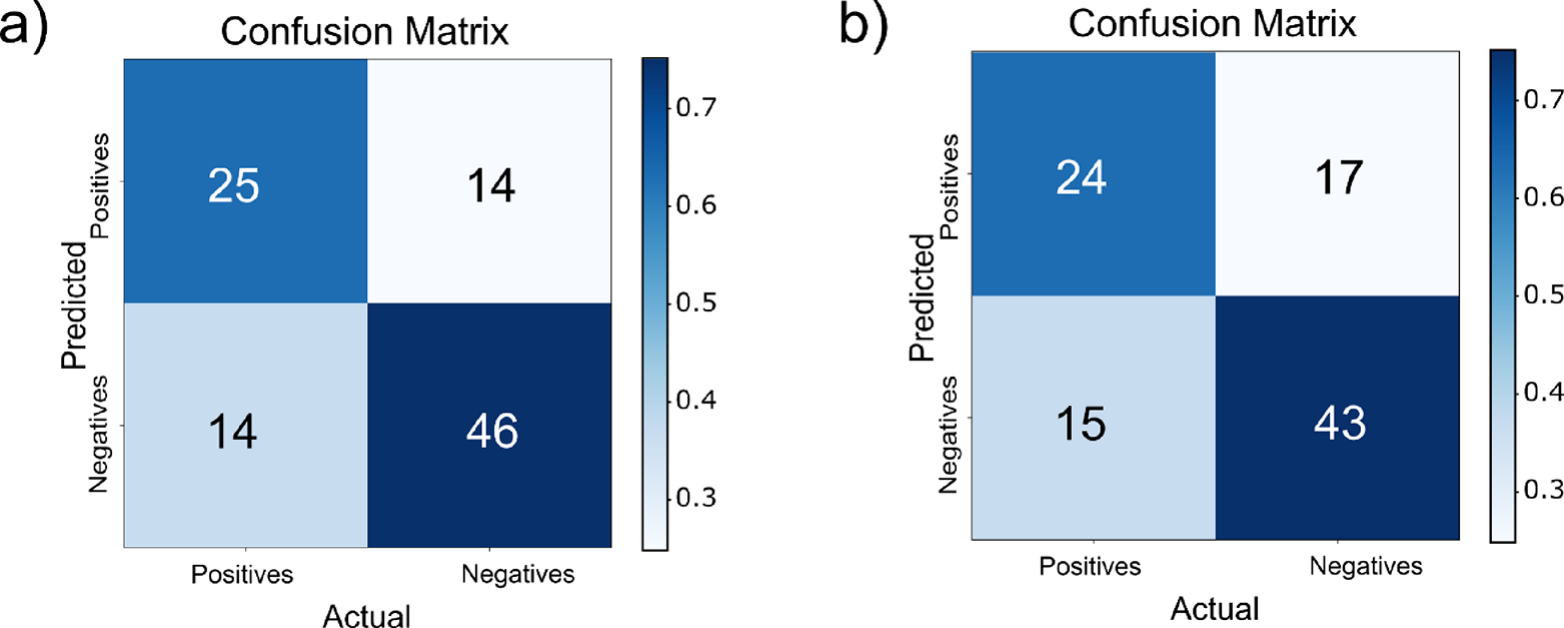
Confusion matrices for test set predictions of UPEC models. (a) UPEC-ToF (*p* < 0.0001) and (b) UPEC-Tof + RDKit (*p* < 0.01).

Two models were generated for UPEC attachment. One model used 53 ToF ions, while the other used 24 features (RDKit descriptors and ToF ions). The test set sensitivity, specificity, and G mean were similar, but the more complex model had a better test set MCC, although the difference may not be statistically significant. Although the more complex Model 7 had a higher test MCC, it also had a much larger number of adjustable parameters, so, applying the principle of parsimony, the simpler model is preferred. Both UPEC models (rows 7 and 8 in Table 2) provided statistically significant results, having passed the test with *p* values of <0.0001 and <0.01, respectively (Figure 3a,b).

When both the training and test target variables were shuffled (thus performing a Y scrambling) after using the same filters as those of the best models generated without shuffling, all MCC values were much lower than the scores observed without Y scrambling (Table 2). This strongly suggests that no chance correlations have occurred in the modeling process.

### 3.1 Feature Analysis

**Figure 4:**
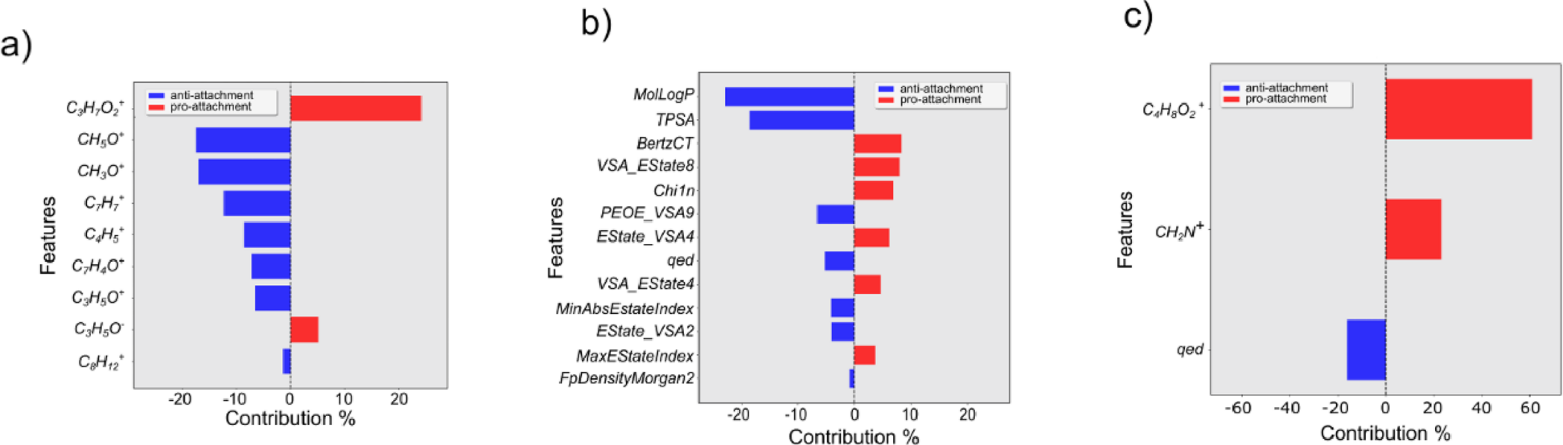
Feature coefficients for test set predictions for PA models. (a) Top 10 feature coefficients for PA-ToF, (b) all 13 feature coefficients for PA-RDKit, and (c) all three feature coefficients for PA-RDKit + ToF.

Feature coefficients for each of the three PA models are shown in Figure 4.

Two of them (Figure 4b,c) used RDKit descriptors either exclusively or in combination with ToF-SIMS ions, while the model in Figure 4a used ToF-SIMS ions only. Regarding the ToF-SIMS ion interpretation, we were able to find a moderate correlation with simple chemoinformatic descriptors for four of them (S-4). Many features of the model in Figure 4 appeared ambiguous and related to common moieties found in many polymers; for example, C_3_H_7_O_2_^+^, CH_3_H_5_O^+^, and CH_3_O^+^ were all associated with the polypropylene or polyethylene glycol repeated block that several monomers in the dataset were made of. Other ToF-SIMS ions, such as C_7_H_7_^+^ and C_8_H_12_^+^, are believed to have come from the polymer backbone. This explained why polyfunctional acrylates, which have multiple polymerization sites and would thus produce a cross-linked polymeric mesh, produced a higher yield for those ions. Finally, an anti-attachment contribution was observed for the acetophenone ion peak (C_7_H_4_O^+^). The C_4_H_8_O_2_^+^ ion, which comes from the repeated polyethylene glycol units, was the most influential peak in the model built from both ToF-SIMS ions and RDKit descriptors (Figure 4c) and has previously been reported to have a strong pro-attachment effect.^22^ The CH_2_N^+^ peak was uniquely present in the only nitrogen-bearing monomer in the dataset, and almost all its polymers were labeled as pro-attachment.

Four descriptors from the RDKit model (Figure 4b) were related to the physicochemical properties of the materials as previously described (calculated log octanol/water partition coefficient MolLog*P*,^41^ topological polar surface area TPSA,^42^ molecular complexity index BertzCT,^43^ and drug-likeness index qed^44^), while others had to be analyzed more carefully (see the Supporting Information). Within the library of monomers used in this study, TPSA is correlated positively with the number of nitrogen and oxygen atoms,^42^ and a strong anti-attachment contribution for this descriptor suggested the importance of such heteroatoms in the monomeric unit. Qed was included both in the RDKit model and the ToF-SIMS ions and RDKit model, showing a moderate anti-attachment effect in both cases and being the only feature in the ToF + RDKit model that can be correlated with simple chemoinformatic descriptors (see the Supporting Information).

**Figure 5:**
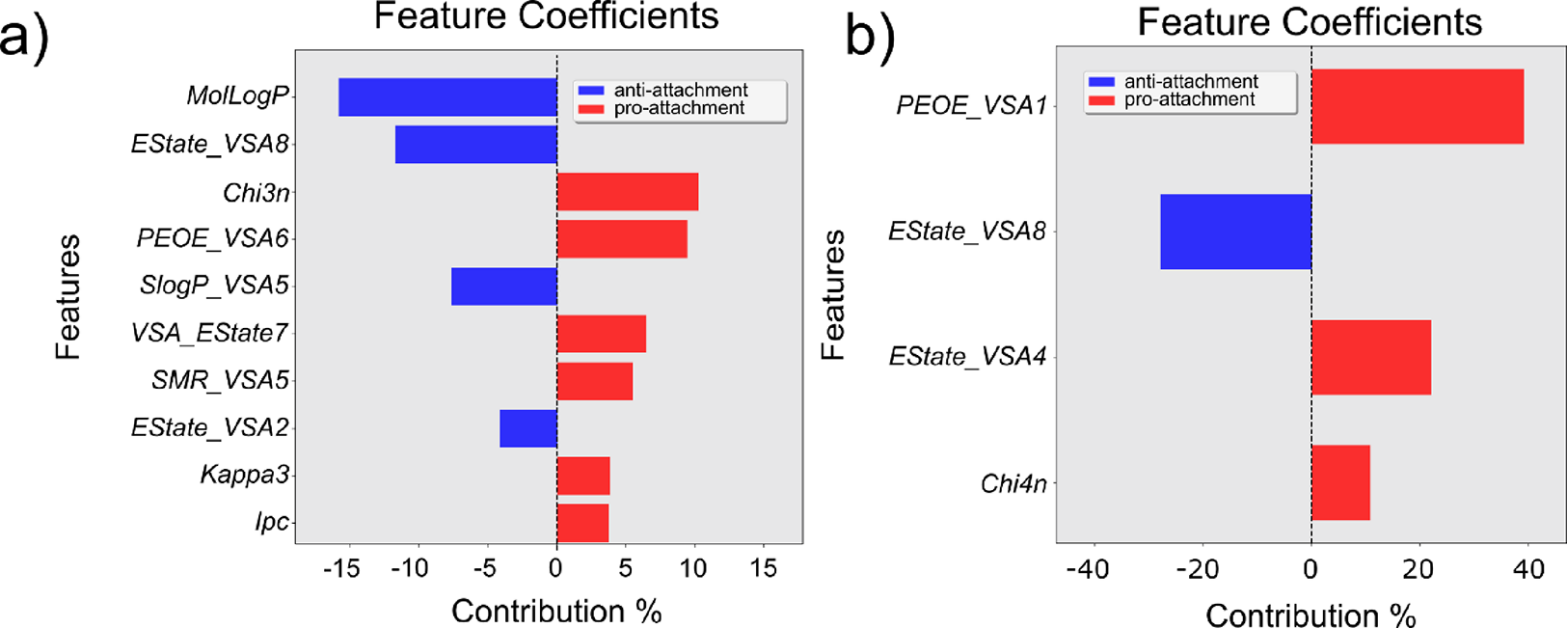
Feature coefficients for test set predictions of SA models. (a) Top 10 feature coefficients for SA-RDKit extended and (b) all four feature coefficients for SA-RDKit simple.

Analysis of the RDKit descriptors in both SA models is shown in Figure 5. The SA extended model used 19 features (Model 5 in Table 2; Figure 5a), while the SA simple model used only four RDKit descriptors (Model 6 in Table 2; see Figure 5b). In the more complex model, as with the model in Figure 1b for PA, MolLog*P* was the most important anti-attachment feature among the top 10 (Figure 5a). All other descriptors in both models (S-4) did not have a clear chemical meaning.

**Figure 6:**
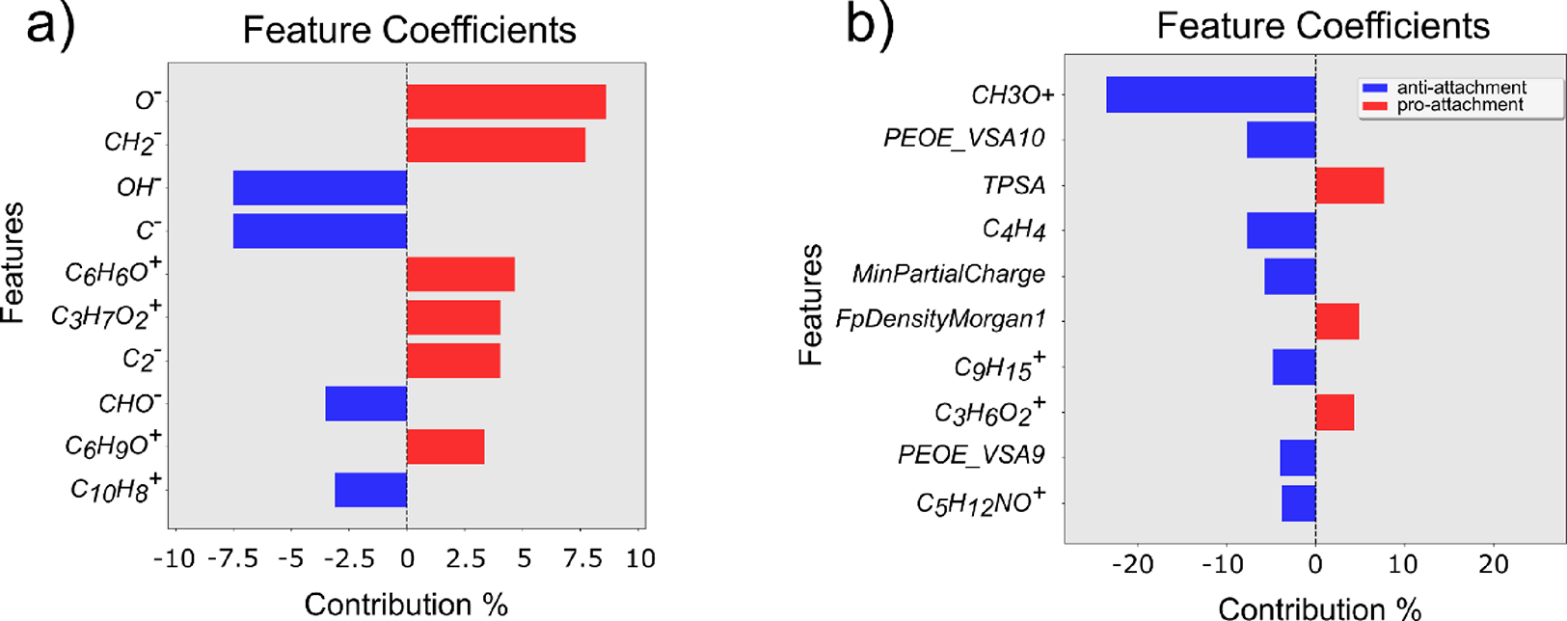
Feature coefficients for test set predictions of UPEC models. (a) Top 10 feature coefficients for UPEC-ToF and (b) top 10 feature coefficients for UPEC–ToF + RDKit.

Finally, we looked at the descriptors in both UPEC models (Figure 6). The first uses 53 ToF ion peaks (Model 7 in Table 2), while the second uses a combination of RDKit descriptors and ToF ion peaks to give a total number of 24 features (Model 8 in Table 2). As was observed for the PA–ToF model, the ubiquitous nature of many peaks that could be found in a wide range of pro- and anti-attachment polymers made the task very difficult. However, the C_6_H_6_O^+^ phenyl ion peak can be easily recognized in Figure 6a, and it shows a moderate pro-attachment effect. Overall, we were able to provide some chemical meaning for two features (S-8).

The interpretation of the top 10 features of the UPEC–ToF + RDKit model (Figure 6b) was assisted by correlating them with interpretable features (see full description in S-8). This is a novel approach to the interpretation of arcane molecular descriptors generated by packages such as Dragon and RDKit. The CH_3_O^+^ ion peak made the largest contribution to the model, having a strong anti-attachment coefficient and being associated with the ethylene glycol and propylene glycol repeated units commonly found in many monomers of the dataset. Interestingly, that ion also had a high anti-attachment coefficient in the PA–ToF model (Figure 4a).

### 3.2 Design Rules for Low Attachment Polymers

Simple design rules for polymers can be very useful to chemists in efficiently creating new materials with desirable antifouling and anti-attachment properties.^45^ After studying the relationship between each model descriptor and the pool of easily interpretable descriptors (the full procedure is available in the Supporting Information), we deduced several simple rules in a decreasing order of importance that defined the main monomer characteristics needed for strong anti-attachment polymers for all three bacteria (Table 3).

**Table 3:** Summary of the Design Rules for Anti-attachment Polymers^a^

rule number	PA	SA	UPEC
1	log *P* > 1.9	N + O < 5	monomer MW > 330 Da
2	rings ≥ 1	monomer MW < 300 Da	aromatic rings = 0
3	rotatable bonds < 11	heteroatoms < 6	–NH or −OH ≥ 1
4	monomer MW < 300 Da	aliphatic carbocycles = 0	rotatable bonds < 12
5	–NH or −OH ≥ 1	log *P* > 2	
6		rotatable bonds < 10	

a The small sets of design rules derived after descriptor interpretation are listed for each bacterium: *P. aeruginosa*, *S. aureus*, and *E. coli*.

Higher lipophilicity and a smaller number of rotatable bonds have previously been reported as crucial parameters for acrylates in resisting PA attachment.^46^ Moreover, lipophilicity, polarity, the number of nitrogen/oxygen atoms, and molecular complexity were also reported to be important in a previous modeling work, which used more complex and non-linear methods.^23^

In SA models, similar to the PA models, we observed a preferred threshold for the minimum accepted log *P* and maximum tolerated monomeric molecular weight as well as for the number of rotatable bonds, although their importance was lower. However, the major factor was the need to have <5 nitrogen and/or oxygen atoms. This requirement contradicts the third rule that a good monomer should ideally have >6 heteroatoms, although this characteristic is applicable to a broad set of elemental constituents and is consistent with the strong anti-attachment behavior of fluorinated polymers in the dataset. A smaller number of nitrogen and oxygen atoms for achieving improved anti-bacterial properties is consistent with the literature for SA.^22^

As with the PA models and unlike the SA models, amine or hydroxyl groups were permitted in UPEC the models possibly because PA and UPEC are Gram-negative bacteria. A low number of rotatable bonds was a consistent rule for all three bacterial species, suggesting that rigid pendant groups might play a role in modulating bacterial attachment, regardless of other structural differences between PA, SA, and UPEC. This is consistent with previous studies that suggest a role of molecular rigidity for achieving resistance to bacterial attachment.^46,47^

## 4 Conclusions

Adhesion of bacteria to biomedical devices is a serious and growing problem due to the ever-increasing numbers of implanted devices used that promote biofilm-centered infections, biofilm tolerance to antibiotic therapy, and the problem of multi-antibiotic resistance. Prevention rather than treatment of infection is a key challenge for medical research. Discovery of new materials supporting very low bacterial attachment and biofilm inhibition is an important strategy to reduce mortality associated to bacterial infections and ease the economic burden on national healthcare systems. Here, we have shown that a binary classification approach can predict the attachment behavior of PA, SA, and UPEC on polyacrylates with good statistical significance when trained using ToF ions, RDKit chemoinformatic descriptors, or a combination of both. An important outcome of the study is the ability to provide design rules for anti-attachment monomers, which was achieved through feature analysis that enabled a simplified interpretation of the model. The results identified the particular importance of moderate to high lipophilicity (log *P* > 2) and a small number of rotatable bonds (<10–12). These play a key role for PA attachment and can be extended to SA and possibly UPEC despite SA being a Gram-positive species with a structurally different cell envelope compared with PA and UPEC. The presence of electronegative or hydrogen bond donor–acceptor nitrogen and/or oxygen functionalities also supported the low attachment for PA and UPEC but enhanced SA adhesion, which is consistent with literature. SA attachment was also modulated by the presence of fluorine atoms. The models generated in this study and the generalized design rules established will be useful for the future design and development of novel anti-bacterial materials. Notably, the models generated using computed molecular descriptors only can be used to virtually screen many potential monomers (near the domain of applicability of the models) to identify new polymers with improved anti-attachment properties.

## Data Availability

All relevant datafiles and codes are available at DOI:10.17639/nott.7256.

## Supporting Information Available

Explanation of datasets; explanation of the Python code and its application to the datasets; explanation of how the diversity filter was devised and applied; list of descriptors used by all models shown in Table 2; list of simple descriptors used for feature interpretation; feature interpretation method; and feature interpretation results (PDF)

## Abbreviations

HAIs: healthcare-associated infections
SSIs: surgical site infection
UTIs: urinary tract infections
ECM: extracellular polymeric matrix
QSAR: quantitative structure–activity relationship
PLS: partial least squares
ML: machine learning
SA: *Staphylococcus aureus*
PA: *Pseudomonas aeruginosa*
UPEC: uropathogenic *Escherichia coli*
BRANN: Bayesian regularized neural network
ToF-SIMS: time-of-flight single ion mass spectrometry
GFP: green fluorescent protein
SFS: sequential forward selection
LR: logistic regression
MCC: Matthew’s correlation coefficient
RMSE: root mean squared error
TPSA: topological polar surface area
