# Supplementary material for: Linear Binary Classifier to Predict Bacterial Biofilm Formation on Polyacrylates
Source: ACS Appl Mater Interfaces. 2023 Mar 7;15(11):14155–63. doi: 10.1021/acsami.2c23182 (PMC10037238; doi:10.1021/acsami.2c23182)
Supplement: Supplementary file 1 — am2c23182_si_001.pdf [file am2c23182_si_001.pdf]

A linear, binary classifier to predict bacterial biofilm formation on polyacrylates

Leonardo Contreas[a], Andrew L. Hook[a], David A. Winkler[a,b,c], Grazziela Figueredo[d], Paul Williams[e], Charles A. Laughton[a], Morgan R. Alexander[a] and Philip M. Williams[a]

[a] School of Pharmacy, University of Nottingham, NG7 2RD, Nottingham, United Kingdom

[b] Monash Institute of Pharmaceutical Sciences, Monash University, Parkville, Australia

[c] School of Biochemistry and Genetics, La Trobe Institute for Molecular Science, La Trobe University, Bundoora, Australia

[d] School of Computer Science, University of Nottingham, Nottingham NG8 1BB, United Kingdom

[e] National Biofilms Innovation Centre, Biodiscovery Institute and School of Life Sciences, University of Nottingham, Nottingham, NG7 2RD United Kingdom.

Information about OS, software, and main libraries used:

Code, available at doi:10.17639/nott.7256, was written in Python (3.7.4) in the Jupyter Notebook (5.7.0) development environment running under Microsoft Windows 10 Enterprise. Molecular

descriptors were from the RDkit (2019.9.3) open-source cheminformatics software suite (www.rdkit.org). All other libraries used were part of the Anaconda (www.anaconda.com) distribution. These could be installed separately using `conda` or `pip3`.

Datasets:

Original datasets used in this study (Microsoft Excel files of ToF ion peak intensities and bacterial attachment data provided in `3 – Datasets and modelling/Original datasets`) were preprocessed and cleaned. For the 4.1c dataset, the Python `Outlier Pruning for classification.ipynb` notebook removes problematic replicates.

Molecular descriptors:

Molecular structures in .mol format, provided in the `1 – Monomer structures` folder, are used by the Python notebook `2 – Descriptor generation/Descriptor_generator.ipynb` to produce Excel files of molecular descriptors. These files can be used alongside ToF-SIMS ion peak data files for both polyacrylate libraries as descriptor sources for modelling. A dataset is ready for modelling once it is in a tabular form, with as many rows as the number of chemical species/polymers, and as many columns as the number of descriptors/features obtained via experimental procedures (ToF-SIMS ion peaks) or via any chemoinformatic software (rdkit library, Dragon software). A final column should be represented by the y-response that we want the model to learn and predict. Such y-response is supposed to be a vector of categorical values for classification tasks.

Modelling:

The `3 - Datasets and modelling\ML analysis.ipynb` contains, step-by-step, the tasks and procedures for the modelling of the data.

Each code block is thoroughly commented to provide as many details as possible on the procedures performed and how code can be changed to meet the researcher's needs. Each code block can be run independently from the others, as long as the variable assignment logic is respected (i.e.: a cell containing a task performed by some function cannot be run if the cell containing the definition of such function has not been run before)

Diversity filter: algorithm and implications

Shannon's entropy is a measure of the amount of information carried by a single feature, and low entropy means a low level of information, as it measures the uncertainty of the outcome during a sampling process [1,2] . We chose to measure a descriptor diversity as the ratio between Shannon's entropy of the descriptor vector and the Shannon's entropy of an ideal descriptor vector, whose values are equally distributed. For example:

$$d_{real} = [1,1,0,0,0,0,0,0,0,0]$$
$$d_{ideal} = [1,1,1,1,1,0,0,0,0,0]$$

where d_{real} is the descriptor vector with all its actual values, and d_{ideal} is the descriptor vector in which all values have the same frequency.

$$S_{d_{real}} = -\sum_{i=1}^{n} P(x_{real_i}) lnP(x_{real_i}) = -(0.2\ln(0.2)) - (0.8\ln(0.8)) = 0.321 + 0.178 = 0.499 \quad (2)$$

$$S_{d_{ideal}} = -\sum_{i=1}^{n} P(x_{ideal_i}) lnP(x_{ideal_i}) = -(0.5\ln(0.5)) - (0.5\ln(0.5)) = 2 * 0.347 = 0.694 \quad (3)$$

$$Diversity = \frac{S_{dreal}}{S_{dideal}} = \frac{0.499}{0.694} = 0.719 \quad (4)$$

where $P(x)$ is the frequency of the x-th value in the descriptor vector, $S_{d_{real}}$ is the Shannon's entropy of the descriptor vector, $S_{d_{real}}$ is the Shannon's entropy of the ideal descriptor vector, and D is the diversity associated to the descriptor vector. In this particular example, the feature would be accepted if the diversity threshold was set to 0.7, but it would have been rejected if the threshold was higher.

List of descriptors used for PA – model 1 (Table2): ['qed', 'MaxEStateIndex', 'PEOE_VSA6', 'MinPartialCharge', 'Ipc', 'EState_VSA4', 'PEOE_VSA7', 'VSA_EState5', 'Kappa3', 'FpDensityMorgan1', 'PEOE_VSA9', 'VSA_EState8', 'MolLogP', 'MolWt', 'VSA_EState1', 'BertzCT', 'MinEStateIndex', 'MinAbsEStateIndex', 'Chi2n', 'SMR_VSA5', 'FractionCSP3', 'VSA_EState7']

List of descriptors used for PA – model 2 (Table2): ['qed', 'Chi1n', 'VSA_EState8', 'PEOE_VSA9', 'MolLogP', 'BertzCT', 'EState_VSA4', 'FpDensityMorgan2', 'MinAbsEStateIndex', 'TPSA', 'MaxEStateIndex', 'EState_VSA2', 'VSA_EState4']

List of descriptors used for PA – model 3 (Table2): ['qed', 'CH_2N', 'C_4H_8O_2']

List of descriptors used for PA – model 4 (Table2): ['C_4H_5', 'C_7H_7', 'C_3H_7O_2', 'CH_5O', 'C_8H_12', 'C_3H_5O[-]', 'C_7H_4O', 'CH_3O', 'C_3H_5O']

List of descriptors used for SA – model 5 (Table2): ['TPSA', 'BalabanJ', 'VSA_EState7', 'EState_VSA8', 'PEOE_VSA9', 'MaxPartialCharge', 'FractionCSP3', 'SlogP_VSA5', 'Chi3n', 'Ipc', 'Kappa3', 'EState_VSA4', 'SMR_VSA5', 'PEOE_VSA7', 'EState_VSA2', 'MinPartialCharge', 'FpDensityMorgan2', 'PEOE_VSA6', 'MolLogP']

List of descriptors used for SA – model 6 (Table2): ['PEOE_VSA1', 'EState_VSA4', 'EState_VSA8', 'Chi4n']

List of descriptors used for UPEC – model 7 (Table2): ['C_6H_8O', 'C[-]', 'C_9H_16', 'CHO_2', 'CH_2', 'C_3H_2[-]', 'C_10H_8', 'C_5H_7', 'C_3H_3O', 'C_13H_9O', 'C_5H_6O', 'C_2H_3O_3', 'C_2H_3', 'C_6H_8O_2', 'C_5H_12N', 'NH[-]', 'C_6H_12', 'O[-]', 'C_3H_7O_2', 'C_5H_9', 'C_7H_9', 'C_4H_8O', 'C_4H_6O', 'C_6H_6O', 'C_9H_9', 'C_4H_6O_2', 'C_6H_9O', 'C_4H_3O', 'C_3H_2O', 'C_5H_9O', 'C_5H_12NO', 'C_3H_6', 'O_2H[-]', 'C_9H_13', 'C_9H_7', 'C_7H_13', 'CH_2[-]', 'C_8H_12', 'C_2[-]', 'CHO[-]', 'C_12H_13', 'OH[-]', 'C_4H_5O', 'CH_2N', 'C_4H_9O', 'C_4H_5O_2[-]', 'C_5H_8O', 'C_7H_6', 'C_4H_8O_2', 'C_2H', 'C', 'C_3H_2', 'C_2H_3O_2']

List of descriptors used for UPEC – model 8 (Table2): ['FpDensityMorgan1', 'C_3H_2[-]', 'C_3H_7O_2', 'C_5H_11O_2', 'C_4H_6O', 'MinPartialCharge', 'C_9H_16', 'TPSA', 'CH_3O_2', 'PEOE_VSA10', 'C_5H_5', 'C_3H', 'C_9H_15', 'PEOE_VSA9', 'C_4H_8O_2', 'C_10H_14', 'C_4H_6', 'C_3H_6O_2', 'C_3HO_2', 'PEOE_VSA7', 'C_4H_4', 'C_3H[-]', 'C_5H_12NO', 'CH_3O']

Feature Analysis: list of simple descriptors used to interpret model features

['MolWt','HeavyAtomMolWt','NumValenceElectrons','NumRadicalElectrons','HeavyAtomCount', 'NHOHCount','NOCount','NumAliphaticCarbocycles','NumAliphaticHeterocycles', 'NumAliphaticRings','NumAromaticCarbocycles','NumAromaticHeterocycles','NumAromaticRings,

'NumHAcceptors','NumHDonors','NumHeteroatoms','NumRotatableBonds', 'NumSaturatedCarbocycles','NumSaturatedHeterocycles','NumSaturatedRings','RingCount', 'MolLogP']

Feature Analysis: mapping simple descriptors to arcane descriptors used by the models

Of interest was the ability to use the models created to identify a set of general rules regarding the chemical properties of the materials being used that would inform the *ab initio* design of biofilm-resistant polymers. As molecular descriptors are often complex and arcane, we correlated each of the top 10 descriptors selected by the models (all 13 features were investigated for the PA model using RDKit descriptors, Model 2 of Table2 in the main text) with simpler and chemically relevant descriptors such as rotatable bonds and molecular weight to understand the underlying chemical-biological interactions governing the material performance. The initial 22 simple descriptors were autoscaled and a polynomial expansion up to the 3rd degree used to broaden the feature space and access to new combined descriptors, generating a total of 2299 composite descriptors. That is, the 22 simple descriptors were combined with each other, or with themselves, twice. After setting maximum tolerated cross-correlation to 0.8, the number of combined features were reduced to 27. Then, we computed all the $\binom{27}{k}$ combinations, with k=1, k=2 and then k=3, to obtain a total number of 2951 different feature subsets, made of a linear combination of up to three original or composite auxiliary descriptors. Subsequently, a linear regression model was trained on the dataset using one

feature subset at a time, and model performance was assessed through the evaluation of Pearson's R^2 and Root Mean Squared Error ($RMSE$). Then, a 10-fold cross-validation was carried out and its average $RMSE_{cv}$ was also computed to assess model robustness (more details are available in Supporting Information). Since the target descriptor was standardized, its standard deviation was equal to 1, thus, the performance of the model could be assessed from its $RMSE_{cv}$. In fact, the $RMSE$ can be seen as the standard deviation of prediction residuals, representing the uncertainty on future predictions[3]. So, if the predicted target variable showed an $RMSE_{cv}$ lower than the standard deviation of the target variable we concluded the model was performing better than random by a $1/RMSE_{cv}$ factor.

Despite the use of cross-validation, phenomena of chance correlation cannot be ruled out. According to a study from Topliss and Costello[4], the risk of observing chance correlation between two independent variables randomly taken from a pool of descriptors, and the target variable, progressively reduces with the number of training samples. For example, for 85 observations and 30 descriptors, the maximum R^2 value that two out of those 30 descriptors (thus $\binom{30}{2}$ combinations) can generate by chance is estimated to be 0.40. Our total number of feature subsets was calculated to be 2299, which is roughly comparable to $\binom{54}{2}$ different combinations. The maximum tolerated R^2 obtainable by chance cannot be directly estimated for this case, but we are confident that a high number of training samples (ranging from 144 for PA in the c496 dataset to 472 for UPEC in

c496+h106 dataset), as well as rejecting any observed correlation if $R^2 < 0.6$, considerably lower the risk of chance correlation in the descriptor interpretation process.

Feature Analysis: interpretation of features of model 4 in Table 2 of the main text (PA – ToF)

- $C_3H_7O_2^+ = MolWt(1.01 - 0.59MolLogP^2) + 0.28NHOHCount$ $[R^2 = 0.66, RMSE_{cv} = 0.61]$
- $CH_3O^+ = MolWt(0.81 - 0.25NumAromaticCarbocycles * NumRotatableBonds) - 0.23MolLogP^2 * NumSaturatedRings$ $[R^2 = 0.71, RMSE_{cv} = 0.54]$
- $C_4H_5^+ = -0.57MolWt + 0.43NumSaturatedRings * MolLogP^2 - 0.33NHOHCount * NumAromaticCarbocycles$ $[R^2 = 0.63, RMSE_{cv} = 0.66]$
- $C_3H_5O^+ = 0.89MolWt + NumAromaticCarbocycles(0.36NHOHCount - 0.33)$ $[R^2 = 0.78, RMSE_{cv} = 0.46]$

Feature Analysis: interpretation of features of model 2 in Table 2 of the main text (PA – RDKit)

- $MolLogP =$ logarithm of the octanol-water partition coefficient[5]
- $TPSA = MolWt - 0.49MolLogP - 0.13NumAliphaticCarbocycles$ $[R^2 = 0.97, RMSE_{cv} = 0.21]$; it is the sum of the contribution of the Van der Waals area of all polar atoms (such as oxygens and nitrogens), and therefore it is also affected by the lipophilicity of the molecule[6].
- $BertzCT = 0.81MolWt + NumAromaticCarbocycles(0.58 - 0.27NHOHCount)$ $[R^2 = 0.90, RMSE_{cv} = 0.37]$; it is a molecular complexity index[7].
- $VSA_{Estate8} = 0.61MolWt - 0.31NHOHCount * MolLogP - 0.30 NumAromaticCarbocycles$ $[R^2 = 0.69, RMSE_{cv} = 0.55]$
- $Chi1n = MolWt(0.91 + 0.11MolLogP^2) + 0.11MolLogP^2 * NumSaturatedRings$ $[R^2 = 0.99, RMSE_{cv} = 0.11]$
- $PEOE\_VSA9 = MolWt(1.31 - 1.22MolLogP^2) - 0.23MolLogP^2 * NHOHCount$ $[R^2 = 0.71, RMSE_{cv} = 0.55]$

- $EState\_VSA4 = MolWt$ $(0.79MolLogP^2 - 0.24NumAromaticCarbocycles * NumRotatableBonds)$ -0.31 $NumAromaticCarbocycles)$ $[R^2 = 0.75, RMSE_{cv} = 0.53)$
- $qed = MolWt(-1.25 + 06MolLgP^2 + 0.31NHOHCount * RingCount)$ $\left[R^2 = 0.83, RMSE_{cv} = 0.46\right]$. It can be defined as a "drug-likeness" descriptor[8], providing a new way to look at how a molecule fits within Lipinski's rule of 5[9].
- $EState\_VSA2 = MolWt(-1.18MolLogP^2 + 0.98)$ $+0.17NHOHCount *$ $NumAliphaticCarbocycles$ $\left[R^2 = 0.64, RMSE_{cv} = 0.62\right]$
- $FpDensityMorgan2 = -0.55MolWt$ $+0.43NumAliphaticCarbocycles$ $+0.37$ $NHOHCount * NumAromaticCarbocycles$ $[R^2 = 0.76, RMSE_{cv} = 0.47]$

Feature Analysis: interpretation of features of model 5 in Table 2 of the main text (SA – RDKit extended)

- $MolLogP$[5]
- $Chi3n = 0.71NOCount$ $+0.6NumAliphaticCarbocycles$ $+0.59MolLogP^2$ $\left[R^2 = 0.94, RMSE_{cv} = 0.26\right]$
- $SLogP\_VSA5 = -2.14MolWt$ $+1.97NOCount$ $+1.94MolLogP^2$ $\left[R^2 = 0.64, RMSE_{cv} = 0.66\right]$
- $SMR\_VSA5 = 0.93MolLogP$ $+0.43NHOHCount$ $-00.35NumAromaticCarbocycles$ $\left[R^2 = 0.71, RMSE_{cv} = 0.55\right]$
- $Kappa3 = NOCount(0.43MolLogP + 0.61)$ $-0.26MolWt * RingCount$ $\left[R^2 = 0.89, RMSE_{cv} = 0.41\right]$
- $PEOE\_VSA9 = NOCount(-0.63MolLogP + 0.71)$ $+0.41Molwt$ $\left[R^2 = 0.60, RMSE_{cv} = 0.70\right]$
- $TPSA = 0.94NOCount$ $+0.17NHOHCount$ $-0.04MolWt$ $[R^2 = 0.99, RMSE_{cv} = 0.13]$ [6]
- $BalabanJ = 0.54NumHeteroatoms^2$ $-0.55MolWt * RingCount$ -0.11 $NumAliphaticCarbocycles * MolLogP^2$ $\left[R^2 = 0.73, RMSE_{cv} = 0.53\right]$; it measures the molecular connectivity [10].
- $PEOE\_VSA7 = 0.72NumAliphaticCarbocycles$ $+0.54NOCount * MolLogP$ $+0.17$ $NHOHCount$ $\left[R^2 = 0.74, RMSE_{cv} = 0.53\right]$
- $FpDensityMorgan2 = 0.75NOCount$ $-0.42NumHeteroatoms^2$ $+0.23NHOHCount$ $[R^2 = 0.74, RMSE_{cv} = 0.56]$

Feature Analysis: interpretation of features of model 6 in Table 2 of the main text (SA – RDKit simple)

- $PEOE_{VSA1} = 0.96 NOCount + 0.04 NumHeteroatoms^2 - 0.04$ $NumAliphaticCarbocycles * NumRotatableBonds^2$ [$R^2 = 0.95, RMSE_{cv} = 0.23$]
- $EState_{VSA4} = 0.57 NOCount * MolLogP - 0.54 NumHeteroatoms^2 + 0.48 MolWt$ [$R^2 = 0.66, RMSE_{cv} = 0.57$]
- $Chi4n = 0.81 NumAliphaticCarbocycles + 0.58 NOCount + 0.45 MolLogP$ [$R^2 = 0.96$, $RMSE_{cv} = 0.21$]

Feature Analysis: interpretation of features of model 7 in Table 2 of the main text (UPEC – ToF)

- $C_6H_6O^+ = NHOHCount * NumAromaticCarbocycles$ $(0.92 + 0.08 NumAliphaticCarbocycles) - 0.06 NHOHCount$ [$R^2 = 0.86, RMSE_{cv} = 0.49$]
- $C_2^- = 1.74 NumAromaticCarbocycles + NumRotatableBonds$ $(0.86 NumAliphaticRings - 1.18 RingCount)$[$R^2 = 0.71, RMSE_{cv} = 0.57$]

Feature Analysis: interpretation of features of model 8 in Table 2 of the main text (UPEC – ToF+RDKit)

- $C_3HO^+ = 0.75 MolWt - 0.4 NumAromaticCarbocycles + 0.28 NHOHCount$ [$R^2 = 0.73$, $RMSE_{cv} = 0.55$]
- $PEOE\_VSA10 = 0.77 NumAromaticCarbocycles + NHOHCount$ $(-0.18 MolLogP^2 + 0.55)$[$R^2 = 0.75, RMSE_{cv} = 0.51$
- $TPSA = 1.16 MolWt - 0.6 MolLogP + 0.03 NumAliphaticCarbocycles$ [$R^2 = 0.94, RMSE_{cv} = 0.25$]
- $C_4H_4^+ = 2.29 NumAromaticCarbocycles + NumRotatableBonds$ $(1.56 NumAliphaticRings - 2.23 RingCount)$ [$R^2 = 0.61, RMSE_{cv} = 0.65$
- $FpDensityMorgan1 = -1.12 MolWt + 0.6 MolLogP + 0.42 NHOHCount$ [$R^2 = 0.77$, $RMSE_{cv} = 0.51$]

References

(1) Jost, L. Entropy and Diversity. *Oikos* **2006**, *113* (2), 363–375. https://doi.org/10.1111/j.2006.0030-1299.14714.x.

(2) Bonaccorso, G. *Machine Learning Algorithms*; Packt, 2017.

(3) Alexander, D. L. J.; Tropsha, A.; Winkler, D. A. Beware of R2: Simple, Unambiguous Assessment of the Prediction Accuracy of QSAR and QSPR Models. *Journal of Chemical Information and Modeling* **2015**, *55* (7), 1316–1322. https://doi.org/10.1021/acs.jcim.5b00206.

(4) Topliss, J. G.; Costello, R. J. Change Correlations in Structure-Activity Studies Using Multiple Regression Analysis. *Journal of medicinal chemistry* **1972**, *15* (10), 1066–1068. https://doi.org/10.1021/jm00280a017.

(5) Comer, J.; Tam, K. Lipophilicity Profiles: Theory and Measurement. *Pharmacokinetic Optimization in Drug Research* **2007**, 275–304. https://doi.org/10.1002/9783906390437.ch17.

(6) Prasanna, S.; Doerksen, R. Topological Polar Surface Area: A Useful Descriptor in 2D-QSAR. *Current Medicinal Chemistry* **2008**, *16* (1), 21–41. https://doi.org/10.2174/092986709787002817.

(7) Bertz H., S. The First General Index of Molecular Complexity. *Journal of the American Chemical Society* **1981**, *103* (12), 3599–3601.

(8) Bickerton, G. R.; Paolini, G. V.; Besnard, J.; Muresan, S.; Hopkins, A. L. Quantifying the Chemical Beauty of Drugs. *Nature Chemistry* **2012**, *4* (2), 90–98. https://doi.org/10.1038/nchem.1243.

(9) Lipinski, C. A.; Lombardo, F.; Dominy, B. W.; Feeney, P. J. Experimental and Computational Approaches to Estimate Solubility and Permeability in Drug Discovery and Development Settings. *Advanced Drug Delivery Reviews* **1996**, *23*, 3–26. https://doi.org/10.1016/j.addr.2012.09.019.

(10) Balaban, A. T. Highly Discriminating Distance-Based Topological Index. *Chemical Physics Letters* **1982**, *89* (5), 399–404. https://doi.org/10.1016/0009-2614(82)80009-2.
